# *N*-(2-Pyridylmethyleneamino)dehydro­abietylamine

**DOI:** 10.1107/S1600536809009040

**Published:** 2009-03-19

**Authors:** Yong Wu, Xiao-Ping Rao, Zong-De Wang, Zhan-Qian Song, Xu-Jie Yao

**Affiliations:** a Institute of Chemical Industry of Forest Products, Chinese Academy of Forestry, Nanjing 210042, People’s Republic of China; b College of Landscape Architecture and Art, Jiangxi Agriculture University, Nanchang 330045, People’s Republic of China

## Abstract

The title compound {systematic name: 1-[(1*R*,4a*S*,10a*R*)-7-isopropyl-1,2,3,4,4a,9,10,10a-octa­hydro­phenanthren-1-yl]-*N*-[(*E*)-2-pyridylmethyleneamino]methanamine}, C_26_H_33_N_2_, has been synthesized from dehydro­abietylamine. The two cyclo­hexane rings form a *trans* ring junction with classic chair and half-chair conformations, respectively, whereas the benzene and pyridine rings are almost planar, and the dihedral angle between them is 80.4°. The two methyl groups directly attached to the tricyclic nucleus are on the same side of the tricyclic hydro­phenanthrene structure.

## Related literature

For the biological activity of a related compound, , see: Cannon (1952 ▶); Heinrich (1981 ▶); Kalser & Scheer (1976 ▶); Rao, Song & He (2008 ▶); Rao, Song, He & Jia (2008 ▶); Wilkerson *et al.* (1991 ▶, 1993 ▶). For the crystal structure of a related compound, see: Rao *et al.* (2006 ▶, 2007 ▶); Rao, Song, Jia & Shang (2008 ▶).
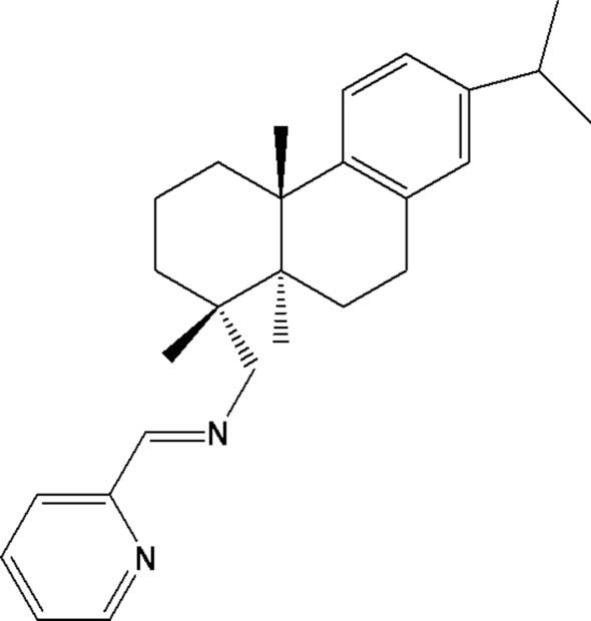


## Experimental

### 

#### Crystal data

C_26_H_33_N_2_
*M* *_r_* = 373.54
Monoclinic, 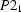

*a* = 11.294 (2) Å
*b* = 6.0870 (12) Å
*c* = 16.129 (3) Å
β = 98.71 (3)°
*V* = 1096.0 (4) Å^3^
*Z* = 2
Mo *K*α radiation
μ = 0.07 mm^−1^
*T* = 293 K
0.30 × 0.20 × 0.10 mm

#### Data collection

Enraf–Nonius CAD-4 diffractometer
Absorption correction: ψ scan (North *et al.*, 1968 ▶) *T*_min_ = 0.951, *T*_max_ = 0.974
2478 measured reflections
2357 independent reflections
1434 reflections with *I* > 2σ(*I*)
*R*_int_ = 0.045
3 standard reflections every 200 reflections intensity decay: none

#### Refinement

*R*[*F* ^2^ > 2σ(*F* ^2^)] = 0.064
*wR*(*F* ^2^) = 0.188
*S* = 1.00
2357 reflections
253 parameters
1 restraint
H-atom parameters constrained
Δρ_max_ = 0.19 e Å^−3^
Δρ_min_ = −0.20 e Å^−3^

### 

Data collection: *CAD-4 Software* (Enraf–Nonius, 1989 ▶); cell refinement: *CAD-4 Software*; data reduction: *XCAD4* (Harms & Wocadlo, 1995 ▶); program(s) used to solve structure: *SHELXS97* (Sheldrick, 2008 ▶); program(s) used to refine structure: *SHELXL97* (Sheldrick, 2008 ▶); molecular graphics: *SHELXL97*; software used to prepare material for publication: *SHELXL97*.

## Supplementary Material

Crystal structure: contains datablocks I, global. DOI: 10.1107/S1600536809009040/at2704sup1.cif

Structure factors: contains datablocks I. DOI: 10.1107/S1600536809009040/at2704Isup2.hkl

Additional supplementary materials:  crystallographic information; 3D view; checkCIF report

## Figures and Tables

**Fig. 1.**
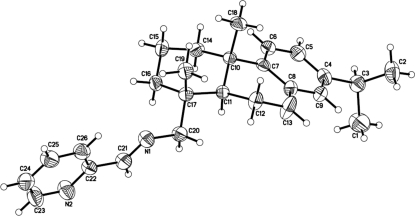
The molecular structure of the title compound, with H atoms represented by small spheres of arbitrary radius and displacement ellipsoids at the 30% probability level.

## supplementary crystallographic information

### Comment

Dehydroabietylamine is a highly interesting compound for its special structure and wide range of applications (Rao, Song, He & Jia, 2008). As an excellent chiral resolving agent, dehydroabietylamine is successful applied in the coalescent of Penicillin (Cannon,1952) and the synthesis of dihydroxyphenylalanine (Kalser *et al.*, 1976). Dehydroabietylamine derivatives exhibited broad spectrum of biological properties including antibacterial, antifungal, and antipenetrant activities (Heinrich, 1981; Wilkerson *et al.*, 1991; Wilkerson *et al.*, 1993; Rao *et al.*, 2007; Rao, Song, & He, 2008; Rao, Song, Jia & Shang, 2008)). Although much attention has been paid to dehydroabietylamine derivatives, the crystal structure of the title compound has not yet been reported. In this paper, we present the crystal structure of the title compound.

The title structure is compared with previously found structure 4-chloro-2-{(*E*)-[(1*R*,4aS,10aR)-7-isopropyl-1,4a-dimethyl-1,2, 3,4,4a,9,10,10*a*-octahydrophenanthren-1-yl] methyliminomethyl} phenol (Rao *et al.*, 2006). They exhibited the same configurations with each other. As shown in Fig.1, the title compound contains four crystrallographically rings, the two cyclohexane rings (rings C and B) form a *trans* ring junction with classic chair and half-chair conformations, respectively. The benzene ring and the pyridine ring (rings A and D) are almost planar. The two methyl groups directly attached to the tricyclic nucleus are on the same side of the tricyclic hydrophenanthrene structure, and the two methyl groups are in the axis position of the cyclohexane ring, the bond lengths and bond angles in the molecule are in normal ranges.

### Experimental

The title compound was prepared by the reaction of dehydroabietylamine (0.1 mol) and pyridylaldehyde (0.1 mol) in ethanol (100 ml) under 353.5 K for 4 h. Single crystals of the title compound were obtained by solvent evaporation [m.p. 372K].

### Refinement

H atoms were positioned geometrically and refined as riding atoms, with C—H = 0.96Å and *U*_iso_(H) = 1.5*U*_eq_(C) for methyl H atoms, and C—H = 0.97–0.98Å and *U*_iso_(H) = 1.2*U*_eq_(C) for all other H atoms. The high Flack value was resulted by the crystal quality.

### Crystal data

**Table:**

C_26_H_33_N_2_	*F*(000) = 406
*M**_r_* = 373.54	*D*_x_ = 1.132 Mg m^−^^3^
Monoclinic, *P*2_1_	Mo *K*α radiation, λ = 0.71073 Å
Hall symbol: P 2yb	Cell parameters from 25 reflections
*a* = 11.294 (2) Å	θ = 10–13°
*b* = 6.0870 (12) Å	µ = 0.07 mm^−^^1^
*c* = 16.129 (3) Å	*T* = 293 K
β = 98.71 (3)°	Block, white
*V* = 1096.0 (4) Å^3^	0.30 × 0.20 × 0.10 mm
*Z* = 2	

### Data collection

**Table:**

Enraf–Nonius CAD-4 diffractometer	1434 reflections with *I* > 2σ(*I*)
Radiation source: fine-focus sealed tube	*R*_int_ = 0.045
graphite	θ_max_ = 26.0°, θ_min_ = 1.3°
ω/2θ scans	*h* = 0→13
Absorption correction: ψ scan (North *et al.*, 1968)	*k* = 0→7
*T*_min_ = 0.951, *T*_max_ = 0.974	*l* = −19→19
2478 measured reflections	3 standard reflections every 200 reflections
2357 independent reflections	intensity decay: none

### Refinement

**Table:**

Refinement on *F*^2^	Primary atom site location: structure-invariant direct methods
Least-squares matrix: full	Secondary atom site location: difference Fourier map
*R*[*F*^2^ > 2σ(*F*^2^)] = 0.064	Hydrogen site location: inferred from neighbouring sites
*wR*(*F*^2^) = 0.188	H-atom parameters constrained
*S* = 1.00	*w* = 1/[σ^2^(*F*_o_^2^) + (0.1*P*)^2^] where *P* = (*F*_o_^2^ + 2*F*_c_^2^)/3
2357 reflections	(Δ/σ)_max_ < 0.001
253 parameters	Δρ_max_ = 0.19 e Å^−^^3^
1 restraint	Δρ_min_ = −0.20 e Å^−^^3^

### Special details

**Table:**

Geometry. All e.s.d.'s (except the e.s.d. in the dihedral angle between two l.s. planes) are estimated using the full covariance matrix. The cell e.s.d.'s are taken into account individually in the estimation of e.s.d.'s in distances, angles and torsion angles; correlations between e.s.d.'s in cell parameters are only used when they are defined by crystal symmetry. An approximate (isotropic) treatment of cell e.s.d.'s is used for estimating e.s.d.'s involving l.s. planes.
Refinement. Refinement of *F*^2^ against ALL reflections. The weighted *R*-factor *wR* and goodness of fit *S* are based on *F*^2^, conventional *R*-factors *R* are based on *F*, with *F* set to zero for negative *F*^2^. The threshold expression of *F*^2^ > σ(*F*^2^) is used only for calculating *R*-factors(gt) *etc*. and is not relevant to the choice of reflections for refinement. *R*-factors based on *F*^2^ are statistically about twice as large as those based on *F*, and *R*- factors based on ALL data will be even larger.

### Fractional atomic coordinates and isotropic or equivalent isotropic displacement parameters (Å^2^)

**Table:**

	*x*	*y*	*z*	*U*_iso_*/*U*_eq_	
N1	0.2116 (4)	0.1781 (8)	−0.1368 (2)	0.0616 (12)	
N2	0.0270 (5)	0.4047 (9)	−0.3125 (3)	0.0814 (15)	
C1	0.6341 (7)	0.9761 (12)	0.3876 (4)	0.108 (3)	
H1B	0.5647	1.0519	0.3598	0.162*	
H1C	0.6861	0.9407	0.3477	0.162*	
H1D	0.6758	1.0684	0.4307	0.162*	
C2	0.7016 (5)	0.6441 (12)	0.4723 (4)	0.088 (2)	
H2B	0.6750	0.5139	0.4975	0.132*	
H2C	0.7436	0.7369	0.5151	0.132*	
H2D	0.7543	0.6041	0.4333	0.132*	
C3	0.5953 (5)	0.7653 (10)	0.4268 (3)	0.0653 (15)	
H3A	0.5469	0.8099	0.4695	0.078*	
C4	0.5153 (5)	0.6250 (9)	0.3642 (3)	0.0560 (14)	
C5	0.3996 (4)	0.5769 (10)	0.3732 (3)	0.0602 (15)	
H5A	0.3690	0.6303	0.4197	0.072*	
C6	0.3280 (4)	0.4524 (9)	0.3157 (3)	0.0541 (13)	
H6A	0.2495	0.4261	0.3239	0.065*	
C7	0.3671 (4)	0.3638 (8)	0.2458 (3)	0.0455 (11)	
C8	0.4870 (4)	0.4045 (9)	0.2362 (3)	0.0519 (12)	
C9	0.5572 (4)	0.5346 (10)	0.2954 (3)	0.0604 (14)	
H9A	0.6360	0.5623	0.2883	0.072*	
C10	0.2857 (4)	0.2215 (8)	0.1825 (3)	0.0444 (11)	
C11	0.3278 (4)	0.2466 (8)	0.0964 (2)	0.0433 (10)	
H11A	0.3290	0.4054	0.0865	0.052*	
C12	0.4589 (4)	0.1745 (10)	0.1029 (3)	0.0636 (15)	
H12A	0.4658	0.0218	0.1200	0.076*	
H12B	0.4840	0.1863	0.0482	0.076*	
C13	0.5392 (5)	0.3102 (14)	0.1640 (3)	0.092 (2)	
H13A	0.6185	0.3356	0.1576	0.110*	
C14	0.1547 (4)	0.2973 (9)	0.1759 (3)	0.0534 (13)	
H14A	0.1260	0.2672	0.2284	0.064*	
H14B	0.1507	0.4547	0.1666	0.064*	
C15	0.0736 (4)	0.1820 (11)	0.1046 (3)	0.0639 (15)	
H15A	0.0739	0.0251	0.1152	0.077*	
H15B	−0.0078	0.2349	0.1022	0.077*	
C16	0.1160 (4)	0.2251 (9)	0.0222 (3)	0.0538 (13)	
H16A	0.1099	0.3814	0.0106	0.065*	
H16B	0.0629	0.1501	−0.0217	0.065*	
C17	0.2442 (4)	0.1517 (8)	0.0188 (3)	0.0482 (12)	
C18	0.2950 (5)	−0.0156 (9)	0.2187 (3)	0.0686 (16)	
H18A	0.2672	−0.0171	0.2721	0.103*	
H18B	0.3769	−0.0633	0.2256	0.103*	
H18C	0.2466	−0.1128	0.1808	0.103*	
C19	0.2519 (5)	−0.1014 (9)	0.0118 (3)	0.0617 (15)	
H19A	0.2274	−0.1678	0.0604	0.093*	
H19B	0.3329	−0.1432	0.0082	0.093*	
H19C	0.2002	−0.1500	−0.0376	0.093*	
C20	0.2829 (4)	0.2563 (10)	−0.0602 (3)	0.0568 (13)	
H20A	0.2753	0.4147	−0.0571	0.068*	
H20B	0.3665	0.2223	−0.0615	0.068*	
C21	0.1584 (5)	0.3134 (10)	−0.1878 (3)	0.0612 (14)	
H21A	0.1654	0.4626	−0.1756	0.073*	
C22	0.0852 (4)	0.2423 (10)	−0.2665 (3)	0.0559 (13)	
C23	−0.0394 (6)	0.3452 (14)	−0.3848 (4)	0.094 (2)	
H23A	−0.0807	0.4544	−0.4176	0.113*	
C24	−0.0503 (6)	0.1341 (14)	−0.4132 (4)	0.085 (2)	
H24A	−0.0981	0.1010	−0.4638	0.102*	
C25	0.0104 (6)	−0.0273 (12)	−0.3659 (4)	0.0780 (18)	
H25A	0.0054	−0.1723	−0.3843	0.094*	
C26	0.0784 (5)	0.0259 (11)	−0.2916 (3)	0.0672 (15)	
H26A	0.1196	−0.0824	−0.2582	0.081*	

### Atomic displacement parameters (Å^2^)

**Table:**

	*U*^11^	*U*^22^	*U*^33^	*U*^12^	*U*^13^	*U*^23^
N1	0.074 (3)	0.052 (3)	0.055 (2)	−0.001 (2)	0.000 (2)	−0.007 (2)
N2	0.091 (4)	0.069 (4)	0.079 (3)	0.008 (3)	−0.008 (3)	0.003 (3)
C1	0.152 (7)	0.067 (5)	0.095 (5)	−0.038 (5)	−0.014 (4)	−0.001 (4)
C2	0.084 (4)	0.077 (5)	0.092 (4)	−0.001 (4)	−0.027 (3)	−0.013 (4)
C3	0.073 (4)	0.058 (4)	0.062 (3)	−0.005 (3)	0.003 (3)	−0.007 (3)
C4	0.067 (3)	0.047 (3)	0.049 (3)	0.006 (3)	−0.006 (2)	−0.003 (2)
C5	0.066 (3)	0.063 (4)	0.051 (3)	0.004 (3)	0.008 (3)	−0.001 (3)
C6	0.056 (3)	0.052 (3)	0.054 (3)	−0.001 (3)	0.005 (2)	0.007 (3)
C7	0.055 (3)	0.037 (3)	0.043 (2)	0.002 (2)	0.004 (2)	0.009 (2)
C8	0.050 (3)	0.053 (3)	0.051 (3)	−0.002 (3)	0.001 (2)	−0.005 (3)
C9	0.049 (3)	0.066 (4)	0.064 (3)	−0.012 (3)	0.002 (2)	0.005 (3)
C10	0.044 (2)	0.033 (3)	0.054 (2)	−0.001 (2)	0.003 (2)	0.009 (2)
C11	0.046 (2)	0.033 (2)	0.050 (2)	−0.001 (2)	0.0013 (19)	0.001 (2)
C12	0.049 (3)	0.075 (4)	0.066 (3)	−0.001 (3)	0.006 (2)	−0.016 (3)
C13	0.049 (3)	0.146 (7)	0.083 (4)	−0.026 (4)	0.020 (3)	−0.055 (5)
C14	0.050 (3)	0.052 (3)	0.060 (3)	0.000 (2)	0.013 (2)	0.004 (3)
C15	0.043 (2)	0.078 (4)	0.069 (3)	−0.001 (3)	0.000 (2)	0.001 (3)
C16	0.049 (3)	0.047 (3)	0.061 (3)	0.000 (3)	−0.005 (2)	0.005 (3)
C17	0.055 (3)	0.030 (2)	0.059 (3)	−0.002 (2)	0.005 (2)	−0.001 (2)
C18	0.087 (4)	0.046 (3)	0.068 (3)	−0.012 (3)	−0.002 (3)	0.019 (3)
C19	0.067 (3)	0.041 (3)	0.073 (3)	−0.003 (3)	−0.002 (3)	−0.004 (3)
C20	0.067 (3)	0.050 (3)	0.053 (3)	−0.008 (3)	0.005 (2)	−0.002 (3)
C21	0.068 (3)	0.054 (3)	0.061 (3)	−0.001 (3)	0.008 (3)	−0.006 (3)
C22	0.061 (3)	0.053 (3)	0.053 (3)	0.002 (3)	0.007 (2)	0.006 (3)
C23	0.089 (5)	0.088 (6)	0.094 (5)	0.014 (4)	−0.021 (4)	0.016 (5)
C24	0.087 (4)	0.098 (6)	0.065 (4)	−0.029 (4)	−0.001 (3)	0.001 (4)
C25	0.102 (5)	0.063 (4)	0.067 (4)	−0.014 (4)	0.008 (3)	−0.005 (4)
C26	0.076 (4)	0.060 (4)	0.064 (3)	−0.005 (3)	0.007 (3)	0.003 (3)

### Geometric parameters (Å, °)

**Table:**

N1—C21	1.251 (6)	C12—H12A	0.9700
N1—C20	1.449 (6)	C12—H12B	0.9700
N2—C23	1.337 (8)	C13—H13A	0.9300
N2—C22	1.345 (7)	C14—C15	1.528 (7)
C1—C3	1.523 (9)	C14—H14A	0.9700
C1—H1B	0.9600	C14—H14B	0.9700
C1—H1C	0.9600	C15—C16	1.501 (6)
C1—H1D	0.9600	C15—H15A	0.9700
C2—C3	1.502 (8)	C15—H15B	0.9700
C2—H2B	0.9600	C16—C17	1.524 (6)
C2—H2C	0.9600	C16—H16A	0.9700
C2—H2D	0.9600	C16—H16B	0.9700
C3—C4	1.513 (7)	C17—C20	1.545 (7)
C3—H3A	0.9800	C17—C19	1.548 (7)
C4—C5	1.368 (7)	C18—H18A	0.9600
C4—C9	1.386 (7)	C18—H18B	0.9600
C5—C6	1.364 (7)	C18—H18C	0.9600
C5—H5A	0.9300	C19—H19A	0.9600
C6—C7	1.381 (6)	C19—H19B	0.9600
C6—H6A	0.9300	C19—H19C	0.9600
C7—C8	1.407 (6)	C20—H20A	0.9700
C7—C10	1.534 (6)	C20—H20B	0.9700
C8—C9	1.392 (7)	C21—C22	1.473 (7)
C8—C13	1.498 (7)	C21—H21A	0.9300
C9—H9A	0.9300	C22—C26	1.376 (8)
C10—C14	1.537 (6)	C23—C24	1.363 (10)
C10—C11	1.543 (6)	C23—H23A	0.9300
C10—C18	1.555 (7)	C24—C25	1.363 (9)
C11—C12	1.532 (6)	C24—H24A	0.9300
C11—C17	1.560 (6)	C25—C26	1.361 (8)
C11—H11A	0.9800	C25—H25A	0.9300
C12—C13	1.485 (7)	C26—H26A	0.9300
			
C21—N1—C20	119.6 (5)	C15—C14—H14A	109.2
C23—N2—C22	116.4 (6)	C10—C14—H14A	109.2
C3—C1—H1B	109.5	C15—C14—H14B	109.2
C3—C1—H1C	109.5	C10—C14—H14B	109.2
H1B—C1—H1C	109.5	H14A—C14—H14B	107.9
C3—C1—H1D	109.5	C16—C15—C14	110.5 (4)
H1B—C1—H1D	109.5	C16—C15—H15A	109.5
H1C—C1—H1D	109.5	C14—C15—H15A	109.5
C3—C2—H2B	109.5	C16—C15—H15B	109.5
C3—C2—H2C	109.5	C14—C15—H15B	109.5
H2B—C2—H2C	109.5	H15A—C15—H15B	108.1
C3—C2—H2D	109.5	C15—C16—C17	114.4 (4)
H2B—C2—H2D	109.5	C15—C16—H16A	108.7
H2C—C2—H2D	109.5	C17—C16—H16A	108.7
C2—C3—C4	113.7 (5)	C15—C16—H16B	108.7
C2—C3—C1	110.9 (6)	C17—C16—H16B	108.7
C4—C3—C1	112.2 (4)	H16A—C16—H16B	107.6
C2—C3—H3A	106.5	C16—C17—C20	107.4 (4)
C4—C3—H3A	106.5	C16—C17—C19	111.0 (4)
C1—C3—H3A	106.5	C20—C17—C19	108.9 (4)
C5—C4—C9	116.6 (5)	C16—C17—C11	108.9 (4)
C5—C4—C3	122.3 (5)	C20—C17—C11	107.2 (4)
C9—C4—C3	121.1 (5)	C19—C17—C11	113.1 (4)
C6—C5—C4	121.6 (5)	C10—C18—H18A	109.5
C6—C5—H5A	119.2	C10—C18—H18B	109.5
C4—C5—H5A	119.2	H18A—C18—H18B	109.5
C5—C6—C7	122.8 (5)	C10—C18—H18C	109.5
C5—C6—H6A	118.6	H18A—C18—H18C	109.5
C7—C6—H6A	118.6	H18B—C18—H18C	109.5
C6—C7—C8	116.9 (4)	C17—C19—H19A	109.5
C6—C7—C10	122.0 (4)	C17—C19—H19B	109.5
C8—C7—C10	121.1 (4)	H19A—C19—H19B	109.5
C9—C8—C7	118.9 (4)	C17—C19—H19C	109.5
C9—C8—C13	120.0 (4)	H19A—C19—H19C	109.5
C7—C8—C13	121.1 (4)	H19B—C19—H19C	109.5
C4—C9—C8	123.2 (5)	N1—C20—C17	112.2 (4)
C4—C9—H9A	118.4	N1—C20—H20A	109.2
C8—C9—H9A	118.4	C17—C20—H20A	109.2
C7—C10—C14	110.5 (4)	N1—C20—H20B	109.2
C7—C10—C11	107.9 (3)	C17—C20—H20B	109.2
C14—C10—C11	109.4 (3)	H20A—C20—H20B	107.9
C7—C10—C18	105.9 (3)	N1—C21—C22	121.6 (5)
C14—C10—C18	108.3 (4)	N1—C21—H21A	119.2
C11—C10—C18	114.7 (4)	C22—C21—H21A	119.2
C12—C11—C10	109.6 (4)	N2—C22—C26	122.7 (5)
C12—C11—C17	114.2 (4)	N2—C22—C21	115.0 (5)
C10—C11—C17	117.0 (4)	C26—C22—C21	122.2 (5)
C12—C11—H11A	104.9	N2—C23—C24	123.9 (7)
C10—C11—H11A	104.9	N2—C23—H23A	118.0
C17—C11—H11A	104.9	C24—C23—H23A	118.0
C13—C12—C11	111.9 (5)	C25—C24—C23	118.6 (6)
C13—C12—H12A	109.2	C25—C24—H24A	120.7
C11—C12—H12A	109.2	C23—C24—H24A	120.7
C13—C12—H12B	109.2	C26—C25—C24	119.4 (7)
C11—C12—H12B	109.2	C26—C25—H25A	120.3
H12A—C12—H12B	107.9	C24—C25—H25A	120.3
C12—C13—C8	117.2 (4)	C25—C26—C22	119.0 (6)
C12—C13—H13A	121.4	C25—C26—H26A	120.5
C8—C13—H13A	121.4	C22—C26—H26A	120.5
C15—C14—C10	112.1 (4)		
			
C2—C3—C4—C5	115.6 (6)	C9—C8—C13—C12	−179.5 (6)
C1—C3—C4—C5	−117.5 (6)	C7—C8—C13—C12	0.5 (9)
C2—C3—C4—C9	−63.0 (7)	C7—C10—C14—C15	171.7 (4)
C1—C3—C4—C9	63.9 (7)	C11—C10—C14—C15	53.0 (5)
C9—C4—C5—C6	−2.2 (8)	C18—C10—C14—C15	−72.6 (5)
C3—C4—C5—C6	179.2 (5)	C10—C14—C15—C16	−58.8 (6)
C4—C5—C6—C7	1.1 (8)	C14—C15—C16—C17	58.4 (6)
C5—C6—C7—C8	0.9 (7)	C15—C16—C17—C20	−166.7 (4)
C5—C6—C7—C10	179.1 (5)	C15—C16—C17—C19	74.4 (6)
C6—C7—C8—C9	−1.8 (7)	C15—C16—C17—C11	−50.8 (6)
C10—C7—C8—C9	180.0 (5)	C12—C11—C17—C16	176.9 (4)
C6—C7—C8—C13	178.2 (5)	C10—C11—C17—C16	46.9 (5)
C10—C7—C8—C13	0.0 (8)	C12—C11—C17—C20	−67.1 (5)
C5—C4—C9—C8	1.3 (8)	C10—C11—C17—C20	162.8 (4)
C3—C4—C9—C8	179.9 (5)	C12—C11—C17—C19	52.9 (6)
C7—C8—C9—C4	0.7 (8)	C10—C11—C17—C19	−77.1 (5)
C13—C8—C9—C4	−179.2 (6)	C21—N1—C20—C17	124.8 (5)
C6—C7—C10—C14	32.6 (6)	C16—C17—C20—N1	−63.6 (5)
C8—C7—C10—C14	−149.3 (4)	C19—C17—C20—N1	56.7 (6)
C6—C7—C10—C11	152.2 (4)	C11—C17—C20—N1	179.5 (4)
C8—C7—C10—C11	−29.7 (6)	C20—N1—C21—C22	179.8 (4)
C6—C7—C10—C18	−84.5 (5)	C23—N2—C22—C26	0.3 (9)
C8—C7—C10—C18	93.6 (5)	C23—N2—C22—C21	179.4 (5)
C7—C10—C11—C12	58.8 (5)	N1—C21—C22—N2	175.8 (5)
C14—C10—C11—C12	179.2 (4)	N1—C21—C22—C26	−5.1 (8)
C18—C10—C11—C12	−59.0 (5)	C22—N2—C23—C24	−0.2 (11)
C7—C10—C11—C17	−169.0 (4)	N2—C23—C24—C25	−0.4 (12)
C14—C10—C11—C17	−48.7 (5)	C23—C24—C25—C26	0.9 (10)
C18—C10—C11—C17	73.2 (5)	C24—C25—C26—C22	−0.7 (9)
C10—C11—C12—C13	−60.7 (6)	N2—C22—C26—C25	0.1 (9)
C17—C11—C12—C13	165.7 (5)	C21—C22—C26—C25	−178.9 (5)
C11—C12—C13—C8	29.8 (8)		
